# Clonal Characterization of Rat Muscle Satellite Cells: Proliferation, Metabolism and Differentiation Define an Intrinsic Heterogeneity

**DOI:** 10.1371/journal.pone.0008523

**Published:** 2010-01-01

**Authors:** Carlo A. Rossi, Michela Pozzobon, Andrea Ditadi, Karolina Archacka, Annalisa Gastaldello, Marta Sanna, Chiara Franzin, Alberto Malerba, Gabriella Milan, Mara Cananzi, Stefano Schiaffino, Michelangelo Campanella, Roberto Vettor, Paolo De Coppi

**Affiliations:** 1 Stem Cell Processing Laboratory, Department of Pediatrics, University of Padua, Padova, Italy; 2 Surgery Unit, Institute of Child Health and Great Ormond Street Hospital, University College London, London, United Kingdom; 3 Department of Cytology, Faculty of Biology, University of Warsaw, Warsaw, Poland; 4 Royal Veterinary College, University of London, London, United Kingdom; 5 Endocrine-metabolic Laboratory, Department of Medical and Surgical Sciences, University of Padua, Padova, Italy; 6 Venetian Institute of Molecular Medicine, Padova, Italy; 7 Consortium for Mitochondrial Research, University College London, London, United Kingdom; Universidad Europea de Madrid, Spain

## Abstract

Satellite cells (SCs) represent a distinct lineage of myogenic progenitors responsible for the postnatal growth, repair and maintenance of skeletal muscle. Distinguished on the basis of their unique position in mature skeletal muscle, SCs were considered unipotent stem cells with the ability of generating a unique specialized phenotype. Subsequently, it was demonstrated in mice that opposite differentiation towards osteogenic and adipogenic pathways was also possible. Even though the pool of SCs is accepted as the major, and possibly the only, source of myonuclei in postnatal muscle, it is likely that SCs are not all multipotent stem cells and evidences for diversities within the myogenic compartment have been described both *in vitro* and *in vivo*. Here, by isolating single fibers from rat *flexor digitorum brevis* (FDB) muscle we were able to identify and clonally characterize two main subpopulations of SCs: the low proliferative clones (LPC) present in major proportion (∼75%) and the high proliferative clones (HPC), present instead in minor amount (∼25%). LPC spontaneously generate myotubes whilst HPC differentiate into adipocytes even though they may skip the adipogenic program if co-cultured with LPC. LPC and HPC differ also for mitochondrial membrane potential (ΔΨ_m_), ATP balance and Reactive Oxygen Species (ROS) generation underlying diversities in metabolism that precede differentiation. Notably, SCs heterogeneity is retained *in vivo.* SCs may therefore be comprised of two distinct, though not irreversibly committed, populations of cells distinguishable for prominent differences in basal biological features such as proliferation, metabolism and differentiation. By these means, novel insights on SCs heterogeneity are provided and evidences for biological readouts potentially relevant for diagnostic purposes described.

## Introduction

Satellite cells (SCs) represent a distinct lineage of myogenic progenitors responsible for the postnatal growth, repair and maintenance of skeletal muscle [1]. They were originally characterized on the basis of their unique position in mature skeletal muscle: closely juxtaposed to the surface of myofibers such that the basal lamina surrounding the SCs and its associated myofiber is a continuous [2], [3]. SCs are mitotically quiescent and activated in response to diverse stimuli, including stretching, injury and electrical stimulation [4], [5], [6]. The descendants of activated SCs, called myogenic precursor cells (MPCs), undergo multiple rounds of cell division before fusing with new or existing myofibers. Although the total number of quiescent SCs decreases with age [7], it remains constant over repeated cycles of degeneration and regeneration thus indicating that the steady-state SCs population is maintained by self-renewal [8], [9], [10], [11]. Therefore, SCs fulfill the criteria of adult stem cells and are distinct from MPCs as underlined by biological and biochemical criteria [2], [12]. Initially, SCs were considered unipotent stem cells with the ability of generating a unique specialized phenotype [2], whilst subsequently, it was demonstrated in mice that opposite differentiation towards osteogenic and adipogenic pathways was also possible [13]. Recently, it was also shown that both human and porcine SCs can differentiate under appropriate stimuli into mature adipocytes [14], [15]. However, even though the pool of SCs is accepted as the major, and possibly the only, source of myonuclei in postnatal muscle, it is most likely that SCs are not all multipotent stem cells [16]. Thus, evidences for diversities within the myogenic compartment have been described both *in vitro* and *in vivo* [17], [18]. Alternative sensitivity to high-dose irradiation revealed that at least two populations of SCs are present [19]: they are distinguishable by proliferative and myogenic capacities [20] with a proportion that varies according to the age [21]. Similarly, after bupivacain injection, two SCs subpopulations get activated: committed myogenic precursors and “stem” satellite cells [22], [23], [24]. Intrinsic heterogeneity was indeed evident when the activating sequence of myogenic regulatory factors (MRFs) was exploited [24]. Among others, Myf5 expression has led to the existence of hierarchical subpopulations of SCs [16;17;25]. In particular, SCs have been shown to be composed of about 10% stem cells (Pax7+/Myf5−) and 90% committed myogenic progenitors (Pax7+/Myf5+) [16]. More recently, variation in the expression of various non-specific myogenic markers such as nestin [26], CXCR-4 and b1-integrin [27], and ABCG2 and Syndecan-4 [28] have also been described. Despite the evident heterogeneity, the phenotypical characteristics of these subpopulations were hard to elucidate because their behavior *in vitro* has been difficult to investigate. Using a new experimental maneuver that permits clear and correct isolation of SCs from the fiber of origin, we report, for the first time, that two subpopulations of SCs coexist in fixed proportions on the single fiber: the low proliferative (LPC) and the high proliferative clones (HPC) which show alternative myogenic potential *in vitro* retained also *in vivo*. Intriguingly, although the HPC give spontaneously rise to adipocytes their myogenic potential can be boosted if co-cultured with LPC. Besides assessing the regenerative and proliferative potentials of SCs, we also investigated functional cellular markers attributable to mitochondrial function. Thus, we exploited the mitochondrial membrane potential (ΔΨ_m_) [29], the pathways of ATP production and the rate of Reactive Oxygen Species (ROS) generation discovering that LPC and HPC remarkably differ in every of these parameters accounting for differences in basal cell signaling and metabolism. In this way, we do not just provide experimental evidences for different populations of SCs but also indications for readouts that may lead to applied studies of regenerative medicine.

## Results

### Low and High Proliferative Clones Are Detected in Single Muscle Fibers Cultured in Hanging Drops

**Figure 1:**
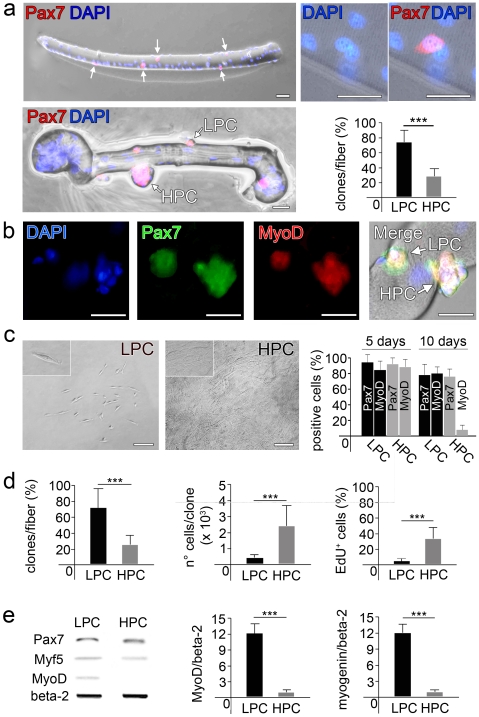
Identification and characterization of 2 subpopulations of SCs. (a) Evaluation of the average number of SCs per single fiber. Isolated FDB single fibers were immunostained with anti-Pax7 antibody and merged with DAPI immediately after isolation, in order to count the SCs (white arrows) per fiber (top panel left; high magnification on top panel right). Single fibers were also cultured in suspension for 5 days and SCs were evaluated. LPC and HPC are distinguishable on the fiber (indicated with LPC and HPC). SCs were immunostained with anti-Pax7 antibody and merged with DAPI (bottom left, bar = 100 µm). In the diagram percentage of LPC and HPC per single fiber (n = 50) is reported (bottom right, mean±s.d., ***p<0.001). (b) Immunostaining for Pax7 and MyoD, merged with DAPI, was performed on both LPC and HPC on fiber, showing that SCs in both clones are positive for such markers after 5 days in suspension culture (bar = 100 µm). (c) Cloning of freshly isolated SCs through limiting dilutions in 96-well dishes. LPC and HPC are distinguishable after phase-contrast microscope analyses (left, bar = 100 µm, insets with higher magnification). In the diagram on the right the % of cells staining positive for Pax7 and MyoD, both in LPC and in HPC, after 5 and 10 days of culture respectively, is reported. (d) In the diagram on the left, percentage of LPC and HPC derived from cloning of freshly isolated SCs per single fiber, then diagrams showing that average number of cells (center) and percentage of EdU-positive cells in LPC and HPC (mean±s.d., ***p<0.001) (right). (e) Semiquantitative PCR analysis for myogenic markers Pax7, Myf5 and MyoD (house-keeping gene: beta-2-microglobulin) (left), and real-time PCR analysis for MyoD (center) and myogenin (right) (mean±s.d., ***p<0.001, normalized against housekeeping gene beta-2-microglobulin) on pools of LPC and HPC.

Single fibers isolated by enzymatic digestion and mechanical dissociation from rat FDB muscles were cultured in hanging drops, technique commonly used for embryonic stem cells culture to avoid cell adhesion and promote cell differentiation [30] (Fig. S1). About 5 Pax7 positive SCs were associated to each single FDB muscle fiber (5.0±0.6, n = 200 fibers) (Fig. 1a, top; Fig. S2) and underwent rapid activation, revealed by MyoD expression and proliferation in culture. In this system proliferating SCs do not leave the native fiber, as normally happens when plated onto a rigid substrate. They remain in their niche under the basal lamina, thus allowing the comparison of different clones arising from each SC. When fibers were maintained in suspension for 5 days, two types of clones were observed on the myofiber surface: low proliferation clones (LPC), containing equal or less than 4 cells, and high proliferation clones (HPC), containing more than 4 cells (Fig. 1a, bottom left). Interestingly, analysis of 70 fibers from 3 different animals revealed that SC clones, identified for the expression of Pax7 and MyoD (Fig. 1b), were present in fixed proportion in each fiber: 3.7±0.9 (about 76%) gave rise to LPC, whilst 1.2±0.6 (about 24%) SCs gave rise to HPC (Fig. 1a, bottom right).

### LPC and HPC Derived from Freshly Isolated SCs

In order to verify that LPC and HPC derived from distinct SC sub-populations, clonal cultures of freshly isolated SCs mechanically disaggregated from single fibers were established. More than 500 fibers out of 10 different animals were analyzed. Purity of stripped cells was confirmed by immunostaining for SC markers 97±1% of stripped cells was positive for Pax7, 92±8% for Myf5 and 95±3% for MyoD after adhesion (500 cells examined for each marker, 10 random fields evaluated at 10× magnification, 5 different experiments; Fig. S3). The absence of contaminating cells in the preparations was confirmed by negative immunostaining for haematopoietic marker CD45, macrophage marker CD163 and endothelial marker CD31 (Fig. S4).

Similarly to what previously reported for other stem cell sources [31], limiting dilutions in 96-well dishes technique was used to determine SC phenotype at single cell level. Single clones maintained in proliferating medium [5] were left for 10 days and examined daily at phase contrast microscope. The number of clones obtained from a single fiber was 2.4±0.7, confirming the absence of contaminant cells (Fig. S2). By phase-contrast microscopy, SC clones were easily distinguished on the basis of their proliferation rate into LPC (Fig. 1c, left) and HPC (Fig. 1c, middle), similarly to what observed in single fiber cultures. A diagram showing Pax7 and MyoD expression in LPC and HPC after 5 and 10 days has been reported (Fig. 1c, right). Moreover, the relative proportion of LPC (1.8±0.4; 75%) and HPC (0.6±0.3; 25%) (Fig. 1d, left) resemble what we have observed with cultures of single fibers (compare with Fig. 1a, bottom right). After 10 days in culture, 50 to 400 cells (323±201) in LPC and 800 to 3.700 (1.432±903) cells in HPC were present (Fig. 1d, center). This was related to a marked difference in the duplication time of LPC and HPC that was 50.4±8 and 18.5±7 hours, respectively. Differences in proliferation were also confirmed by evaluating the amount of EdU (5-ethynyl-2′-deoxyuridine) incorporated by duplicating SCs in an interval of 48 hours. 3±2% of cells in LPC and 29±11% of cells in HPC (p<0.001) were in active DNA synthesis (Fig. 1d, right). For PCR analyses, various LPC and HPC were pooled, in order to collect enough material compatible to the sensibility of the technique. Standard PCR on LPC and HPC showed that both clones expressed Pax7 and Myf5, while the expression of MyoD was not detectable in HPC at day 10 (Fig. 1e, left). At the same time point, quantitative real time PCR analysis confirmed a significant reduction of MyoD expression in HPC (Fig. 1e, center) and showed that myogenin expression was also very low (Fig. 1e, right). Finally, HPC were successfully subcloned using serial dilution technique and they were able to generate LPC and HPC, in a proportion comparable to the one obtained in the first clonal generation (82±16% LPC and 18±10% HPC) (Fig. S5). Heterogeneity of SCs has been extensively described in the last few years. It is believed that SCs, 24 h after activation, express Pax7 and MyoD, but then a proportion of them (around 20%) withdraw from cell cycle to return in a quiescent state (Pax7+ MyoD) that is linked to the maintenance of the SCs pool [32]. This SCs proportion is considered to retain a multipotent differentiation potential [33] but have remained unclear for many years if they represented a distinct population or a dynamic state during SC activation [24]. We found that the proportion of SCs able to proliferate at high rate at clonal level (HPC) was around 25% of the total. Noteworthy, this could plausibly be the subset with stem cell characteristics. Similarly to adult stem cells in fact, they rapidly grow in culture when exposed to proper stimuli, as partly showed before [33], and could be clonally expanded in culture [31] containing high serum.

### SCs Proliferative Potential Correlates with Differences of the F_1_Fo-ATPsynthase Efficiency and ROS Generation Rate

**Figure 2:**
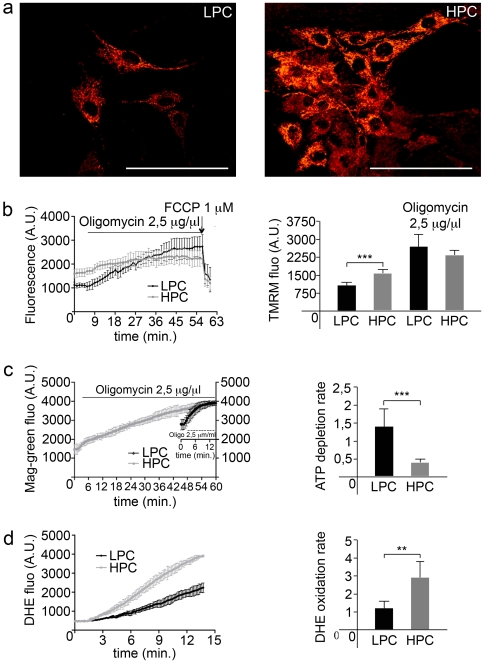
Metabolic characterization of Low and High Proliferative Clones. (a) Confocal images showing TMRM fluorescence as measurement of mitochondrial potential (Δψ_m_). LPC (left) shows, in this culture condition, a lower Δψ_m_ compared to HPC (right). (b) Δψ_m_ was measured in real time in LPC and HPC using TMRM fluorescence and confocal imaging. Cells were treated with oligomycin (2.5 µg/µl) to block the F_O_ component of the F_1_F_O_-ATPasynthase. 1 µM FCCP was added at the end of each experiment to completely depolarize the mitochondria. Diagram on the left shows the trend over time. Values derived from 21 individual cells before and after oligomycin addition for LPC and HPC are summarized in diagram on the right (***p<0.001). (c) Cellular ATP was followed in real time by measuring intracellular [Mg^2+^]_c_ using the fluorescent dye Magnesium Green (Mag-green). LPC and HPC were treated with oligomycin (2.5 µg/µl) and (left) traces trend monitored over time (traces are mean±s.d. of 10 individual experiments for cell type); plotted values of [Mg^2+^]_c_ rate (mean±s.d.) is presented in diagram on the right (***p<0.001). (d) ROS production was measured using 5 µg/ml dihydroethidium (DHE). Diagram on the left shows an increase in DHE fluorescence in LPC and HPC recorded in real time and diagram on the right the rate values (mean±SD) of three different experiments are summarized (**p<0.005).

Intrigued by the differences in proliferation we then investigated whether these two populations of cells might differ also for metabolic markers. Therefore, parameters attributable to the efficiency of mitochondrial function were readily investigated. Assessment of the mitochondrial membrane potential (ΔΨ_m_) with the potentiometric dye tetramethyl rhodamine methyl ester (TMRM) showed that LPC have reduced ΔΨ_m_ than HPC cells (Fig. 2a), suggesting that differences in the coupling of the mitochondrial F_1_Fo-ATPsynthase are likely to occur. Thus, resting ΔΨ_m_ may be a short-hand for protons motive force throughout the Electron Respiratory Chain (ERC) hence a direct function of the F_1_Fo-ATPsynthase in intact cells [34;35] whereas an increased H^+^ flux through the F_1_Fo-ATP synthase reduces the ΔΨ_m_ and conversely a reduced H^+^ flux increases it. To confirm that, clones were subsequently exposed to 2.5 µg/µl oligomycin, a selective inhibitor of the F_1_Fo-ATP synthase activity and the ΔΨ_m_ monitored over time. As a result of an active respiration, the continued efflux of protons increased ΔΨ_m_ (Fig. 2b, left) and this increase was more sustained in LPC than in HPC although both clones eventually reach a final state that normalizes the initial differences (control conditions, LPC: 1121.68±118.95 and HPC: 1595.52±184.18 arbitrary units; after Oligomycin, LPC: 2735.95±499.28 and HPC: 2349.95±224.53 arbitrary units; p<0.001, n = 7 distinct cultures) (Fig. 2b, right).

If this interpretation is correct, the ATP generated from mitochondrial oxidative phosphorylation (OXPHOS) should be the main source of energy in LPC and since these cells could not be transfected with intracellular targeted luciferase [36] (data not shown) to gain a discrete measurement of ATP dynamics, we quantified the concentration of free magnesium [Mg^2+^]_c_ as alternative read-out of ATP fluxes [37] (see Methods). Oligomycin was also employed in this experiment to block the F_1_Fo-ATPsynthase and monitoring the decay of ATP (corresponding to a rise in [Mg^2+^]_c_) in both LPC and HPC. Consistently, the ATP level dropped quite instantly in LPC whilst in HPC a visible effect took over ∼60 minutes to occur (Fig. 2c, left). The kinetic of [Mg^2+^]_c_ was then calculated and plotted as histogram (Fig. 2c, right) (LPC: 1.42±0.49 and HPC: 0.42±0.07 arbitrary units, p<0.001, n = 3 different cultures). These data suggest that HPC metabolism seems to be more glycolytic than that of the LPC in which mitochondria appear more coupled and act as the principal source of intracellular energy. Glycolysis is a feature of every highly proliferative cell (e.g. cancer cells) [38] and also contributed by alterations in fundamental signalling mechanisms the most representative of which is Reactive Oxygen Species (ROS) generation [39], which we have therefore evaluated [40]. This was done using the fluorescent probe dihydroethidium (DHE) which forms a fluorescent product (ethidium) when oxidised allowing a real time detection of basal generation of ROS [41]. The traces reported in Fig. 2d show that in HPC the DHE oxidation is significantly faster than in LPC indicating that the HPC have an increased rate of ROS generation at resting conditions. Values summarized in panel d right (LPC: 1.13±0.41 and HPC: 2.92±0.87; rates from normalized arbitrary units, p<0.005, n = 2 different cultures) confirm that cellular ROS are more prominent in HPC than LPC, consistent data with the findings on ΔΨ_m_ and ATP.

### Spontaneous Adipogenic Differentiation of HPC

**Figure 3:**
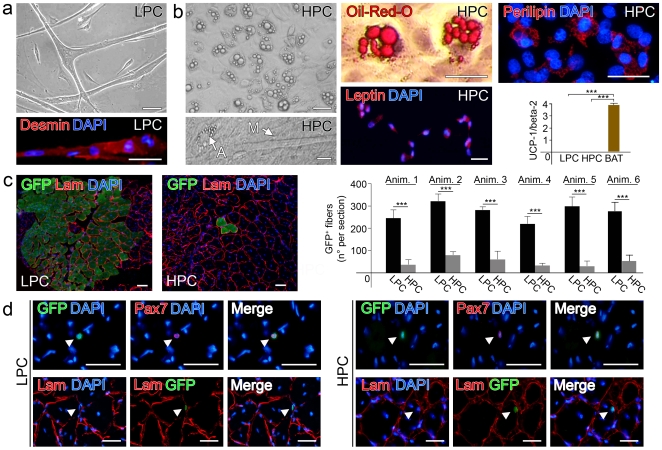
*In vitro* and *in vivo* differentiation potential of subpopulations of SCs. (a) Spontaneous differentiation potential of LPC and HPC in high serum medium: LPC give rise to mature myotubes (top), as visible through phase contrast microscopy, that were positively immunostained for desmin (bottom, bar = 100 µm). (b) HPC give spontaneously rise to adipocytes, well visible through phase contrast microscopy (top left), and a small amount of myotubes (bottom left). Lipid droplets are marked through Oil-Red-O staining (top center) and immunostaining for perilipin (top right) (bar = 100 µm). Immunostaining for leptin on HPC (bottom center). Real-time PCR for mitochondrial uncoupler protein UCP-1 on LPC and HPC, compared with brown adipose tissue (BAT) (***p<0.001; bottom right). (c) Epifluorescence analysis for GFP and immunostaining for laminin on muscle sections injected with LPC (left) and HPC (center). On the right is reported a diagram indicating for each of six animals treated the amount of GFP+ fibers per section of muscle injected with LPC and HPC (mean±s.d.,***p<0.001). (d) Immunofluorescence analysis for Pax7 and epifluorescence for GFP, merged with DAPI on sections of muscles treated with LPC (top panel, left) and HPC (top panel, right). Epifluorescence for GFP and immunostaining for laminin, merged with DAPI, on sections of muscles treated with LPC (lower panel left) and HPC (lower panel right) (bar = 100 µm).

Once dissociated from the parental fiber, LPC and HPC SCs revealed also a distinct differentiation potential. When cultured in myogenic medium, LPC exhibited a normal myogenic differentiation with formation of abundant myotubes (Fig. 3a, top) expressing desmin (Fig. 3a, bottom). Instead, in the same culture conditions, HPC spontaneously formed a large number of multi- and paucilocular adipocytes (Fig. 3b, top left) while generating only rare myotubes (Fig. 3b, bottom left). Adipogenic differentiation was documented by Oil-Red-O staining of lipid droplets (Fig. 3b, top center) and immunodetection of perilipin, an adipocyte-specific protein coating lipid droplets (Fig. 3b, top right). In addition, 97.0±2.8% cells in HPC were positive for leptin (Fig. 3b, bottom center), whereas LPC cells were all negative. Mitochondrial uncoupler protein 1 (UCP-1), a marker of brown adipose tissue (BAT), was not detected by quantitative real time PCR in either LPC or HPC (Fig. 3b, bottom right). Previous studies have demonstrated that SCs can give rise to adipocytes, osteocytes and smooth-muscle cells, other than myotubes, if cultured in conditioned media containing tissue-specific differentiation factors [13], [14]. This was believed to be a stochastic phenomenon driven by the MAD pathway [33]. Interestingly, this property seems to be confined to a fixed proportion of SCs, defined HPC as demonstrated by leptin and perilipin expression and Oil-Red-O staining. Whilst Oil-Red-O is not strictly specific for adipose cells [42], leptin and perilipin expression cannot be found in myogenic cells. Moreover, differently to what previously demonstrated [43], the lipid droplets inside the cytoplasm tend to fuse giving rise to unilocular cells with the morphological features of the white fat cell. This is in accordance with the absence of UCP1 expression in the adipocytes formed by HPCs in these set of experiments as well as in previous studies carried out using both rodents and human SCs [13], [14]. Therefore, we believe that adipogenic potential of SCs at clonal level is limited to a precise proportion of cells that is represented by a subset of those that downregulate MyoD after activation and retain great proliferative potential. Further studies however will be necessary to finally elucidate the fate of adipocytes derived from SCs. In this perspective, one limitation of our study is related to the fact that SCs were derived from one single muscle to avoid the well described intermuscular variability [44].

### LPC Have a Higher Myogenic Regenerative Potential than HPC Following Transplantation In Vivo

In order to investigate whether the remarkable differences of LPC and HPC cells *in vitro* were maintained *in vivo*, pools of 20.000 LPC cells or 20.000 HPC cells, derived from GFP-transgenic rats, were injected separately in regenerating wild-type rat muscles. To induce muscle regeneration, rat *tibialis anterioris* muscles were treated with bupivacaine (which causes muscle fiber necrosis followed by regeneration), injected with the cells 72 hours later and examined after 3 weeks. The contribution of donor cells to myonuclei, determined by the number of GFP positive fibers, was different between LPC and HPC. In particular, the number of GFP-positive muscle fibers was much higher in muscles injected with LPC cells (273±46 per section) than in muscles injected with HPC cells (52±12 per section) (Fig. 3c). On the contrary, the engraftment of donor cells into the SC niche, determined by the presence of GFP positive donor-derived cells in the Pax7 cell population, was not significantly different (Fig. 3d). This may be due to the fact that HPC, which are less committed to myogenic lineages and subjected to high self-renewal, have more chance *in vivo* to engraft in the SC cell niche and could require longer or another cycle of muscle damage to act in myofibers formation. These results are in accordance to what previously reported about the potential of slow-cycling cells *in vitro* that showed a great proliferative potential *in vivo* [17] and have been suggested also by the recent demonstration of a subset of satellite-side population cells which *in vivo* preferentially contribute to the SC-niche [28]. Interestingly, in our setting the number of Pax7+GFP+ cells did not vary among the 2 different groups (Fig. S6). All these data together seem to indicate that while LPC may represent a transplantable population of committed progenitors, both LPC and HPC are functional to maintain SCs niche. In our view this validates once more the high contribution to both muscle regeneration and self-renewal observed when different populations of SCs are transplanted either with their fiber [45] or as freshly isolated cells [46].

### HPC Co-Cultured with LPC Switch from the Adipogenic to the Myogenic Differentiation Program

**Figure 4:**
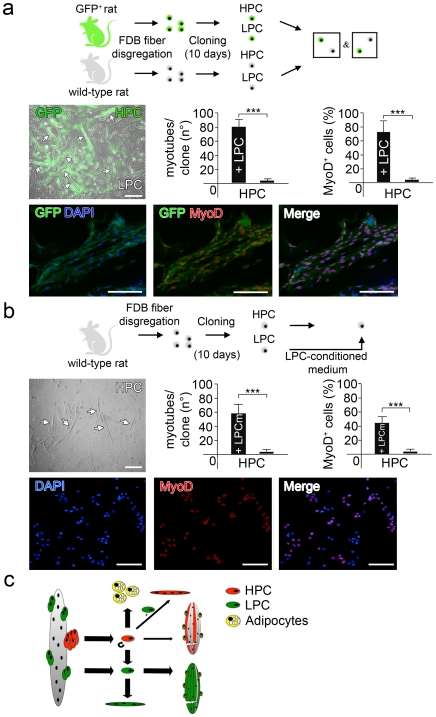
Co-culture of LPC and HPC. (a) Scheme for co-cultures: SCs were derived from FDB muscles of both wild-type and GFP+ rats then cloned through limiting dilutions and after 10 days of culture HPC were seeded on LPC (top panel). Increase in myotube formation derived from HPC. Epifluorescence for GFP+ myotubes (indicated with white arrows, central panel left, bar = 100 µm), and diagrams indicating number of myotubes per clone (central panel, center) and percentage of MyoD+ cells (central panel, right) in co-cultures, compared to control HPC (mean values±s.d., ***p<0.001). Immunostaining for MyoD and epifluorescence for GFP, merged with DAPI on co-cultures (bottom panel, bar = 100 µm). (b) Scheme for culture in conditioned medium: SCs were derived from FDB muscles of wild-type rats then cloned through limiting dilutions. After 10 days of culture LPC-conditioned medium was transferred on HPC (top panel). Myotube formation (indicated with white arrows, central panel left, bar = 100 µm), and diagrams indicating number of myotubes per clone (center) and percentage of MyoD+ cells (right) in cultures with LPC-conditioned medium, compared to control HPC (mean values±s.d., ***p<0.001; middle panel). Immunostaining for MyoD on HPC cells, subjected to LPC-conditioned medium, merged with DAPI (bottom panel, bar = 100 µm). (c) Cartoon of the current working model: pools of Satellite Cells can be isolated from the muscle fiber and cultured in vitro preserving their original diversities. HPC spontaneously differentiate into adipocytes whilst LPC into myoblasts. Notably, HPC may undergo myogenic pathway if cultured together with LPC. LPC and HPC in vitro diversities mirror differences in the capacity of regenerating damaged muscles tissues.

Fat deposition within muscle tissue is not present in physiological conditions and adipocytes are rarely seen in standard SC cultures, suggesting that the propensity of HPC cells to undergo adipogenic differentiation is normally repressed and revealed only when HPC SCs are cultured in isolation. To determine whether this repression is mediated by LPC cells, we designed an experiment in which SCs clones derived from GFP-transgenic and wild type rats were co-cultured. Specifically, we added GFP^+^-HPC to wild-type LPC or wild-type HPC to GFP^+^-LPC, in an average proportion (HPC/LPC) of 26,5 (Fig. 4a, top). Under these conditions, none of the HPC observed gave rise to adipocytes, as determined by phase contrast microscopy, Oil-Red-O staining and immunostaining for perilipin and leptin (data not shown). Instead, we observed an increased tendency of HPC to fuse and differentiate into myotubes (Fig. 4a, middle left). In particular, 20 days after culture 80±9 GFP^+^ myotubes were observed when GFP^+^-HPC were co-cultured with LPC, compared to 3±1 myotubes formed when the same number of HPC were clonally cultured for the same time (Fig. 4a, middle center). The switch from adipogenic to myogenic program was not only induced by fusion of HPC with LPC cells, since also mononucleated HPC cells showed enhanced MyoD expression in the co-cultures. While only 4±2% of HPC cells expressed MyoD in control conditions, MyoD was present in 73±14% of HPC cells after 20 days in co-culture with LPC (Fig. 4a, middle right and bottom). Similarly, when HPC were cultured in LPC-conditioned medium (Fig. 4b, top) a much greater number of myotubes (43±8 vs. 3±1) was formed, and the expression of MyoD was present in 58±7% of HPC cells (Fig. 4b, middle and bottom).

While in physiological conditions, healthy muscle does not present intramuscular adipose tissue (IMAT), this is rather common in pathological conditions as in familiar partial lipodystrophy, obesity, type 2 diabetes and the metabolic syndrome [47]. IMAT can be present in conditions characterized by an increase in muscle mass, such as in acromegaly [48], but it is particularly evident in conditions characterized by a primary decrease in the muscle mass as in myopaties, aging [49], and sedentary young subjects. These changes in the regional body composition with an increase in the adipose tissue within muscle might also affect insulin sensitivity, glucose and lipid metabolism thus mimicking the normal-weight “metabolically obese” syndrome [50]. In our experiments, SCs were derived from young healthy rats and it was unexpected to find such high proportion of cells that in myogenic conditions were able to spontaneously generate mature adipocytes (Fig. 3b). This phenomenon is originated only by a defined clonally expandable population of cells, namely HPC. By these means, we demonstrate that this regulation may also dependent on a cross-talk among distinct progenitors within the SC population similarly to what happens in other systems [51].

## Discussion

SCs and their heterogeneity have been widely investigated and various reports already described differences in their proliferative and myogenic potentials [20], [21], [22], [23]. Here, we confirmed such differences and showed for the first time that in rat myofibers there is a correlation between SCs proliferation and differentiation potential similarly to that previously reported for other stem cells. Using an innovative technique for single muscle fiber culture in suspension, adapted from embryoid-bodies cultures [30] we observed that not only SCs proliferate at different rates [52] but also that the ratio between clones with high (HPC) and low (LPC) proliferative rate was fixed at single fiber level. Moreover, after mechanical dissociation and cloning through limiting dilution, it was still possible to distinguish an identical ratio of LPC and HPC. Notably, differences in proliferation mirror differences in functional parameters such as mitochondrial membrane potential (ΔΨ_m_), ATP balance and ROS generation. HPC compared to the LPC proved to have a more glycolytic phenotype characterized by increased ΔΨ_m_, reduced mitochondrial generation of ATP and higher rate of ROS production. Abnormal rising of the ΔΨ_m_ at resting conditions defines impairments in the H^+^ transport through the Electron Respiratory Chain (ERC) thus acting as a parameter to assess mitochondrial activity [34]. Since this is normalized via pharmacological inhibition of the terminal enzyme of the ERC (the F_1_F_o_-ATPsynthase) (see Fig. 2b), the oxidative phosphorylation of the HPC can be considered underperforming compared to that of the LPC. Therefore, the mitochondrial ATP generation is also reduced and addition of oligomycin able to promote a mild reduction of intracellular ATP in HPC compared to LPC (Fig. 2c). Alternative efficiency of the mitochondrial coupling was not the only distinctive feature between the two populations since HPC presented also a remarkable higher level of ROS production compared to LPC. ROS is a signal associated to cell differentiation into adipocytes [40] and HPC which spontaneously differentiate into adipocytes (Fig. 3b) show plausibly for this reason an increased rate of basal ROS production.

Taken these evidences together it may envisage that modifications in cell metabolism precede and possibly dictate the nature of the ultimate cellular differentiation. It has been already shown, although in alternative models, how environmental and nutritional conditions impinging on the animal metabolism may affect the number and the growth of muscle fibers [53], [54], thus providing evidences for a bond between metabolism and quality of the muscle progenitors. The link between mitochondrial metabolism and stem cells has been previously proposed but an accurate evaluation of its entity and biological significance never performed despite its potential acknowledged (see [55] and references within). Here, we demonstrate that before the myogenic or adipogenic markers could be detectable, cells undergo remarkable modifications in their mitochondrial function. Future studies are needed to explore the real nature of these homeostatic modifications, however, to the best of our knowledge this as the entire clonal characterization here presented has never been described before and strongly suggests that proliferation and differentiation may be pre-determined and not stochastically activated among SCs [33] whereas the metabolic features may act as early biological read-outs.

These innovative results pair with recent studies that recently described the presence of subsets of cells with stem cells characteristics within the SC compartment. HPC in fact fulfill the definition of multipotent stem cells, meaning they can retain their capacity to differentiate in alternative cell types although deriving from the same embryonic compartment [56]. Similarly to SP-satellite cells [28], skeletal muscle precursors-SMP [27] and muscle stem cells-MuSC [10], HPC can contribute *in vivo* to the SC pool when injected after injury and may divide asymmetrically to produce LPC. However, in our experimental setting HPC, differently to SP-satellite cell, SMP and MuSC, are poorly myogenic when cultured clonally. Noteworthy, their myogenic potential is boosted when cultured in contact with conditioned LPC-media. When co-cultured together, distinguishing one or the other clone type via GFP expression, LPC were able to condition HPC and block their adipogenic conversion alongside an increase in the MyoD expression and myotubes formation. We verified that this conditioning was not due to cell-cell contact, but plausibly to released factors since the effects were obtained after 10 days of HPC culture in LPC conditioned medium (Fig. 4). Further studies however are necessary to define which soluble factors (myokine) and signaling pathways are involved in such cross-talk. If it will be confirmed that HPC represent a source of adipogenic tissue within the skeletal muscle in pathological conditions, the ability of controlling their fate could lead to important implications for therapy. However, while this study deeply investigated *in vitro* the characteristics of LPC and HPC their physiological role *in vivo* remains partially unknown.

By these means, we were able to characterize an intrinsic potential of SCs able to give rise to high and low proliferative clones. The high proliferative spontaneously differentiated to adipocytes but they could still be conditioned towards a massive myogenic differentiation if co-cultured with LPC. This is an insightful notion suggesting that a paracrine effect might occur and it would explain why spontaneous adipogenic generation/differentiation is not present in healthy muscles. Although future work is essential to understand the molecular factors conditioning the “choice” of SCs between LPC and HPC as well as the cross-talk existing between these two subpopulations, this study offers novel insights towards a new level of understanding of muscle regeneration, definition and prevention of fat deposition within the skeletal muscle.

## Methods

### Animals

Three to four month-old Sprague-Dawley wild type rats (Harlan, Indianapolis, USA) and transgenic rats, with expression of the enhanced GFP, under the control of the cytomegalovirus (CMV) enhancer and the chicken beta-actin promoter [57], were used in this study. Animal care and experimental procedures were performed in accordance with “D.L. 27-1-1992, number 116, applicative declaration of Healthy Minister number 8 22-4-1994”.

### Isolation of Single Fibers from Flexor Digitorum Brevis (FDB) Muscle

Single muscle fibers with associated SCs were isolated from FDB muscles as previously described [5]. In brief the hind limb FDB was digested for 3 hours at 37°C in 0.2% (w/v) type I-collagenase (Sigma-Aldrich), reconstituted in DMEM (high-glucose, with L-glutamine, supplemented with 1% penicillin-streptomycin, GIBCO-Invitrogen). Following digestion, the muscle was transferred in plating medium (DMEM low-glucose, 10% HS, 1% penicillin-streptomycin, 0.5% chicken embryo extract, GIBCO-Invitrogen) and gently triturated with a wide-bore pipette to release single myofibers. In each preparation, under phase contrast microscope, 50 single fibers were carefully sucked up through a 100 µl pipette and transferred in a 10 cm-plate containing 10 ml of muscle plating medium (1° dilution). Each single fiber was subsequently transferred in another 10 cm plate containing 10 ml of muscle plating medium (2° dilution). Finally, each fiber was collected into one 50 ml Falcon tube with 1 ml of muscle proliferating medium (3° dilution in DMEM low-glucose, 20% FBS, 10% HS, 1% penicillin-streptomycin, GIBCO-Invitrogen, 0.5% chicken embryo extract, MP Biomedicals). Serial dilution was performed in order to avoid the presence of contaminant cells. An aliquot of each preparation was used to perform immunofluorescence staining for CD45, CD163 and CD31 to further exclude respectively macrophages, haematopoietic and endothelial cells contamination after muscle dissociation (1° dilution), first (2° dilution) and second passage (3° dilution).

### Single Fibers Culture in Suspension in Hanging Drops

Freshly isolated single fibers were transferred with Pasteur pipette to drops of proliferating medium. Each 30 µl drop contained only one single fiber devoid of contaminating cells. Twenty drops were made on the upper part of 10 cm Petri dish (Falcon) which was then turned while the lower part of dish was filled with PBS to prevent evaporation, similarly to what is commonly done for embryoid bodies' cultures [30]. Fibers were cultured for 5 days at 37.5°C, 5% CO_2_ in a humidified tissue culture incubator (Heraeus BDD 6220) and then fixed and immunostained.

### Cloning Satellite Cells from Single Myofibers

Clones of SCs were derived from FDB myofibers of Sprague-Dawley rats (both wild type and GFP-transgenic). For each experiment, 10 myofibers were transferred, after dilution, into a separate tube containing 1 ml DMEM. They were then triturated 20 times using a 18 G needle mounted onto a 1 ml syringe, to disengage SCs [33]. The resulting cell suspension was filtered through a 40 µm cell strainer (Falcon) and diluted with 18.2 ml of muscle proliferating medium, and then dispensed into five 96-well petri dishes in 0.2 ml of growth medium (DMEM low-glucose, 20% FBS, 10% HS, 1% penicillin-streptomycin, GIBCO-Invitrogen, 0.5% chicken embryo extract, MP Biomedicals) with limiting dilution (0.5 cell/well). Dishes were incubated at 37.5°C, 5% CO_2_ in a humidified tissue culture incubator. Clones were then followed counting the amount of cells at 5, 10 and 20 days with inverted-microscope analysis and Bürker counting chamber. Duplication time was assessed through the formula: duplication time (hours) = Δt/log_2_ (number of cells at second count/number of cells at first count), with Δt = interval (in hours) between the first and the second count.

### Immunofluorescence Analyses

Immunofluorescence analysis was conducted on freshly isolated single myofibers, myofibers cultured in suspension in hanging drops, SCs disengaged from fibers but not cloned and SCs cloned with limiting dilutions. Freshly isolated single myofibers were collected in 0.5 ml DMEM, 5% HS in an Eppendorf tube. Fibers and SCs were fixed with 4% paraformaldehyde (PFA; Sigma-Aldrich) in phosphate buffered saline (PBS, GIBCO-Invitrogen), rinsed in PBS and permeabilized with Triton X-100 (Fluka) 0.5% in PBS. After washing, fibers and/or cells were incubated with primary antibodies overnight at 4°C or 1 hour at 37°C. Non specific interactions were blocked with 20% goat serum (Vector). They were then washed and incubated with labeled secondary antibodies for one hour at room temperature. SCs were then mounted with fluorescent mounting medium (DAKO) plus DAPI 100 ng/ml (Sigma-Aldrich). Fibers were collected and moved onto a polilysine microscope slide, and then mounted. Immunofluorescence for Pax7 on isolated single fibers was conducted in order to determine the average number of SCs per fiber. The following primary antibodies were used: mouse anti-rat Pax7 (DSHB, Iowa, dilution 1∶50), rabbit anti-rat Myf5 (Santa Cruz, dilution 1∶50), rabbit anti-rat MyoD (Santa Cruz, dilution 1∶50), rabbit anti-rat desmin (Abcam, dilution 1∶50), mouse anti-rat CD45 (Chemicon, dilution 1∶50), mouse anti-rat macrophages CD163 (Serotec, dilution 1∶50), mouse anti-rat CD31 (Chemicon, dilution 1∶50), rabbit anti-rat leptin (Santa Cruz, dilution 1∶10), rabbit anti-rat perilipin A (Abcam, dilution 1∶500), rabbit anti-rat laminin (Sigma, dilution 1∶100). Secondary antibodies used were Alexa Fluor goat anti-mouse 488, Alexa Fluor donkey anti-mouse 594 and Alexa Fluor chicken anti-rabbit 594 (Molecular Probes).

### Cell Proliferation Assay

For the quantification of cell proliferation we used the Click-iT Edu Breakthrough cell proliferation assay (Invitrogen) based on an analogous of bromodeoxiuridine: EdU (5-ethynyl-2′-deoxyuridine), according to manufacturer's protocol.

### Standard PCR Analysis

PCR analysis has been conducted on pools of LPC and HPC, in order to get enough material for the assay. Total ribonucleic acid (RNA) was extracted using a kit (Rneasy Micro, Qiagen), following the supplier's instructions from SCs of both LPC and HPC. All material collected (due to the little number of total cells obtained from a single cloning) was firstly treated with Dnase and removal reagents (Ambion, USA) before being reverse-transcribed using Superscript II reverse transcriptase (Invitrogen). Primers used are reported in Table S1.

### Real-Time PCR

PCR was carried out using a DNA Engine (Opticon 2 Continuous Fluorescence Detection System; MJ Research). Reactions were performed two times with SYBR Green PCR Master Mix (Applied Biosystems) and 5 to 10 ng of cDNA as previously described [58]. Standard curve was obtained using cDNA derived from LPC and HPC for the MRF MyoD, myogenin and UCP-1. Results were normalized by beta-2-microglobulin mRNA content and reported as arbitrary units ratio.

### Imaging Mitochondrial Membrane Potential

For the majority of experiments, we used tetramethyl rhodamine methyl ester (TMRM, 50 nM, Invitrogen) in “redistribution mode” [29]: the dye was allowed to equilibrate and was present continuously. TMRM distributes between cellular compartments in response to different potentials and, at concentrations ≤50 nM, the fluorescent signal shows a simple relationship with the dye concentration, so that signal intensity maps to mitochondrial potential. TMRM fluorescence intensity was quantified by removing all background signal by “thresholding” and measuring the mean TMRM fluorescence intensity in the pixels containing mitochondria. Thus the signal is independent of mitochondrial mass and only reflects the dye concentration within individual mitochondrial structures. Mitochondrial depolarization is seen as the movement of dye from mitochondria into the cytosol. Experiments were the normalized in response to mitochondrial depolarization by 1 µM carbonyl cyanide 4-(trifluoromethoxy)phenylhydrazone (FCCP), which was done at the end of every experiment.

### [Mg^2+^]_c_ Measurement

For measurements of the free cytosolic Mg^2+^ concentration [Mg^2+^]*_c_* as an index of ATP hydrolysis (since ATP has a higher affinity than ADP for Mg^2+^), the Mg^2+^ sensitive dye Magnesium Green (5 µg/ml; Molecular Probes) (*K_d_* = 1 mM) was used [37]. Fluorescent images were captured on Zeiss 510 LSM confocal microscope equipped with a 40X oil-immersion lens.

### ROS Production Measurements

Coverslips were transferred to small chambers for microscopy. Cells were imaged while bathing in a modified HBSS solution containing (in mM) 156 NaCl, 3 KCl, 2 MgSO4, 1.25 KH2PO4, 2 CaCl2, 10 glucose, and 10 HEPES, pH adjusted to 7.35 with NaOH. Dihydroethidium (DHE; 10 µM) was added immediately before the start of an experiment and remained in the solution for the duration. Images were obtained using a Zeiss 510 LSM confocal microscope equipped with a 40X oil-immersion lens. Excitation was provided by the 543 line of the helium-neon laser line and emitted fluorescence collected >560 nm. In all experiments using DHE, data were collected every 10 s. The rate of DHE oxidation was compared between LPC and HPC cells. The rate was calculated in every cell in a field of view was analyzed and included in the final measurements.

### Oil-red-O Staining for Lipid Droplets

Presence of adipose elements in cell culture was determined by Oil-Red-O staining (Sigma-Aldrich) [59]. The slides were fixed in 10% formalin for 1 hour, washed in deionized water, and air-dried. The cells were incubated with Oil-Red-O staining solution for 15 min, counterstained with Mayer's hematoxylin (pH 4), and rinsed in deionized water.

### In Vivo Experiments

In order to test SCs muscle regeneration potential, a model of muscle damage was tested similarly to what previously described [60]. Six Sprague-Dawley female rats were injected with 150 µl of bupivacain 0.5% (w/v, with adrenalin 5 g/ml) in both tibialis anterioris (TA) muscles. After 3 days a pool of HPC GFP+ SC clones (20000 cells) and a pool of LPC GFP+ SC clones (20000 cells), counted after trypsinization, were resuspended in 50 µl of DMEM, and injected separately in left and right TA muscles respectively. Animals were left for 3 weeks, then they were sacrificed and TA muscles were collected, fixed in PFA 2% for 1 hour and left in sucrose 30% overnight. The following day muscles were frozen in isopentane cooled in liquid nitrogen, and subsequently processed using a criostate (Leica) to produce 10 µm sections for staining. GFP-positive fibers have been counted for each muscle section, and average number of positive fibers for each of the six animals has been evaluated.

### HPC-LPC Co-Cultures and HPC in LPC Conditioned Medium

Twenty single HPC were co-cultured with 20 single LPC. Each HPC has been detached with trypsin and seeded on a single LPC. Every experiment has been performed in a single well of 96-well plate (Falcon). Culture of 20 HPC in LPC-conditioned medium has also been performed. LPC-conditioned medium was prepared filtering the supernatant of culture of LPC with 0.22 µm syringe filters (Nalgene) mounted on 2 ml-syringes (Falcon), in order to avoid any LPC-cell contamination in HPC. LPC-conditioned medium was added to HPC very 2 days for all the period of culture (20 days).

### Microscope and Imaging System

Phase-contrast and hystological analyses were conducted using an inverted microscope (Olympus IX71); immunofluorescence analyses were performed using a direct microscope (Leica B5000). GFP-positive fibers were counted through analysis for GFP epifluorescence.

*Statistical analysis. Data are presented as mean±s.d. Comparison between groups used the t-test assuming two-tailed distribution, with an alpha level of 0.05.*

## Supporting Information

Table S1 The primers were built from information taken from: http://frodo.wi.mit.edu/cgibin/primers3/primer3_wwwcgi web site.
(0.04 MB DOC)

Figure S1 20 drops with a single myofiber were posed on the top of a petri dish. It was then turned, in order to perform a suspension culture in hanging drops. After 5 days SCs emanate from the fiber and clones were easily distinguishable between LPC and HPC.
(0.08 MB DOC)

Figure S2 Isolated FDB fibers were immunostained for Pax7 and DAPI. SCs were counted in order to evaluate the average amount present per single, isolated fiber (as in Fig. 1). Diagram shows the average amount of SCs on fiber (F column) and after fiber disaggregation and isolation (AI column, mean±s.d.).
(0.04 MB DOC)

Figure S3 SCs released from isolated myofibers were seeded on gelatin-coated slides, and then immunofluorescence for the canonical marker Pax7 and the myogenic markers Myf5 and MyoD were performed. Diagram indicates percentage of positive cells (mean Â±s.d.).
(1.03 MB DOC)

Figure S4 Freshly isolated SCs were immunostained for haematopoietic cell marker CD45, macrophage marker CD163 and endothelial marker CD31, at each of the 3 passages of dilution (see Materials and Methods). They resulted negative for all contaminant cell markers immediately after the second passage.
(15.29 MB DOC)

Figure S5 HPC clones (n = 10) were sub-cloned in 96-well dishes with limiting dilutions. After 10 days of culture it was possible to distinguish LPC (left) and HPC (center), the latter with adipogenic potential. Their relative proportion was evaluated and reported in diagram (right, mean Â±s.d., ***p<0.001). In the table below the developmental potential of each single sub-cloning is reported.
(0.88 MB DOC)

Figure S6 When the number of Pax7+/GFP+ cells was evaluated after LPC and HPC injection we could not observe any significant difference as reported in the diagram (mean Â±s.d.).
(0.06 MB DOC)

## References

[1] Seale P, Sabourin LA, Girgis-Gabardo A, Mansouri A, Gruss P (2000). Pax7 is required for the specification of myogenic cells.. Cell.

[2] Bischoff R, Heintz C (1994). Enhancement of skeletal muscle regeneration.. Dev Dyn.

[3] Mauro A (1961). Satellite cell of skeletal muscle fibers.. J Biophys Biochem Cytol.

[4] Appell HJ, Forsberg S, Hollmann W (1988). Satellite cell activation in human skeletal muscle after training: evidence for muscle fiber neoformation.. Int J Sports Med.

[5] Rosenblatt JD, Yong D, Parry DJ (1994). Satellite cell activity is required for hypertrophy of overloaded adult rat muscle.. Muscle Nerve.

[6] Schultz E (1985). Satellite cell in normal, regenerating and dystrophic muscle.. Adv Exp Med Biol.

[7] Schultz E, Lipton BH (1982). Skeletal muscle satellite cells: changes in proliferation potential as a function of age.. Mech Ageing Dev.

[8] Gibson MC, Schultz E (1983). Age-related differences in absolute numbers of skeletal muscle satellite cells.. Muscle Nerve.

[9] Morlet K, Grounds MD, McGeachie JK (1989). Muscle precursor replication after repeated regeneration of skeletal muscle in mice.. Anat Embryol (Berl).

[10] Sacco A, Doyonnas R, Kraft P, Vitorovic S, Blau HM (2008). Self-renewal and expansion of single transplanted muscle stem cells.. Nature.

[11] Schultz E, Jaryszak DL (1985). Effects of skeletal muscle regeneration on the proliferation potential of muscle satellite cells.. Mech Ageing Dev.

[12] Grounds MD, Yablonka-Reuveni Z (1993). Molecular and cell biology of skeletal muscle regeneration.. Mol Cell Biol Hum Dis Ser.

[13] Asakura A, Komaki M, Rudnicki M (2001). Muscle satellite cells are multipotential stem cells that exhibit myogenic, osteogenic, and adipogenic differentiation.. Differentiation.

[14] De Coppi P, Milan G, Scarda A, Boldrin L, Centobene C (2006). Rosiglitazone modifies the adipogenic potential of human muscle satellite cells.. Diabetologia.

[15] Singh NK, Chae HS, Hwang IH, Yoo IM, Ahn CN (2007). Transdifferentiation of porcine satellite cells to adipoblasts with ciglitizone.. J Anim Sci.

[16] Kuang S, Kuroda K, Le Grand F, Rudnicki MA (2007). Asymmetric self-renewal and commitment of satellite stem cells in muscle.. Cell.

[17] Beauchamp JR, Heslop L, Yu DS, Tajbakhsh S, Kelly RG (2000). Expression of CD34 and Myf5 defines the majority of quiescent adult skeletal muscle satellite cells.. J Cell Biol.

[18] Rouger K, Brault M, Daval N, Leroux I, Guigand L (2004). Muscle satellite cell heterogeneity: in vitro and in vivo evidences for populations that fuse differently.. Cell Tissue Res.

[19] Heslop L, Morgan JE, Partridge TA (2000). Evidence for a myogenic stem cell that is exhausted in dystrophic muscle.. J Cell Sci.

[20] Molnar G, Ho ML, Schroedl NA (1996). Evidence for a multiple satellite cell population and a non-myogenic cell type that is regulated differently in regenerating and growing skeletal muscle.. Tissue Cell.

[21] Grounds MD, McGeachie JK (1987). A model of myogenesis in vivo, derived from detailed autoradiographic studies of regenerating skeletal muscle, challenges the concept of quantal mitosis.. Cell Tissue Res.

[22] Rantanen J, Hurme T, Lukka R, Heino J, Kalimo H (1995). Satellite cell proliferation and the expression of myogenin and desmin in regenerating skeletal muscle: evidence for two different populations of satellite cells.. Lab Invest.

[23] Schultz E (1996). Satellite cell proliferative compartments in growing skeletal muscle.. Dev Biol.

[24] Cornelison DD, Wold BJ (1997). Single-cell analysis of regulatory gene expression in quiescent and activated mouse skeletal muscle satellite cells.. Dev Biol.

[25] Cooper RN, Tajbakhsh S, Mouly V, Cossu G, Buckingham M (1999). In vivo satellite cell activation via Myf5 and MyoD in regenerating mouse skeletal muscle.. J Cell Sci.

[26] Day K, Shefer G, Richardson JB, Enikolopov G, Yablonka-Reuveni Z (2007). Nestin-GFP reporter expression defines the quiescent state of skeletal muscle satellite cells.. Dev Biol.

[27] Cerletti M, Jurga S, Witczak CA, Hirshman MF, Shadrach JL (2008). Highly efficient, functional engraftment of skeletal muscle stem cells in dystrophic muscle.. Cell.

[28] Tanaka KK, Hall JK, Troy AA, Cornelison DD, Majka SM (2009). Syndecan-4-expressing muscle progenitor cells in the SP engraft as satellite cells during muscle regeneration.. Cell Stem Cell.

[29] Duchen MR, Surin A, Jacobson J (2003). Imaging mitochondrial function in intact cells.. Methods Enzymol.

[30] Keller GM (1995). In vitro differentiation of embryonic stem cells.. Curr Opin Cell Biol.

[31] De Coppi P, Bartsch G, Siddiqui MM, Xu T, Santos CC (2007). Isolation of amniotic fluid stem cell lines with potential for therapy.. Nat Biotechnol.

[32] Zammit PS, Heslop L, Hudon V, Rosenblatt JD, Tajbakhsh S (2002). Kinetics of myoblast proliferation show that resident satellite cells are competent to fully regenerate skeletal muscle fibers.. Exp Cell Res.

[33] Shefer G, Wleklinski-Lee M, Yablonka–Reuveni Z (2004). Skeletal muscle satellite cells can spontaneously enter an alternative mesenchymal pathway.. J Cell Sci.

[34] Campanella M, Casswell E, Chong S, Farah Z, Wieckowski MR (2008). Regulation of the mitochondrial structure and function by the F_1_F_0_-ATPase inhibitor protein, IF1.. Cell Metab.

[35] Campanella M, Seraphim A, Abeti R, Casswell E, Echave P (2009). IF1, the endogenous regulator of the F_1_F_0_-ATP synthase, defines mitochondrial volume fraction in HeLa cells by regulating autophagy.. Biochim Biophys Acta.

[36] Jouaville LS, Pinton P, Bastianutto C, Rutter GA, Rizzuto R (1999). Regulation of mitochondrial ATP synthesis by calcium: evidence for a long-term metabolic priming.. Proc Natl Acad Sci USA.

[37] Leyssens A, Nowicky AV, Patterson L, Crompton M, Duchen MR (1996). The relationship between mitochondrial state, ATP hydrolysis, [Mg^2+^]i and [Ca^2+^]i studied in isolated cardiomyocytes.. J Physiol.

[38] Zhivotovsky B, Orrenius S (2009). The Warburg effect returns to a cancer stage.. Semin Cancer Biol.

[39] Pelicano H, Carney D, Huang P (2008). ROS stress in cancer cells and therapeutic applications.. Drug Resist Updat.

[40] Aguiari P, Leo S, Zavan B, Vindigni V, Rimessi A (2008). High glucose induces adipogenic differentiation of muscle-derived stem cells.. Proc Natl Acad Sci USA.

[41] Bindokas VP, Jordan J, Lee CC, Miller RJ (1996). Superoxide production in rat hippocampal neurons: selective imaging with hydroethidine.. J Neurosci.

[42] Kinkel AD, Fernyhough Me, Helterline DL, Vierck JL, Oberg KS (2004). Oil-red-O stains non-adipogenic cells: a precautionary note.. Cytotechnology.

[43] Seale P, Bjork B, Yang W, Kajimura S, Chin S (2008). PRDM16 controls a brown fat/skeletal muscle switch.. Nature.

[44] Shinin V, Gayraud-Morel B, Gomes D, Tajbakhsh S (2006). Asymmetric division and cosegregation of template DNA strands in adult muscle satellite cells.. Nat Cell Biol.

[45] Collins CA, Olsen I, Zammit PS, Heslop L, Petrie A (2005). Stem cell function, self-renewal, and behavioral heterogeneity of cells from the adult muscle satellite cell niche.. Cell.

[46] Montarras D, Morgan J, Collins C, Relaix F, Zaffran S (2005). Direct isolation of satellite cells for skeletal muscle regeneration.. Science.

[47] Gallagher D, Kelley DE, Yim JE, Spence N, Albu J (2009). Adipose tissue distribution is different in type 2 diabetes.. Am J Clin Nutr.

[48] Freda PU, Shen W, Heymsfield SB, Reyes-Vidal CM, Geer EB (2008). Lower visceral and subcutaneous but higher intermuscular adipose tissue depots in patients with growth hormone and insulin-like growth factor I excess due to acromegaly.. J Clin Endocrin Metab.

[49] Hilton TN, Tuttle LJ, Bohnert KL, Mueller MJ, Sinacore DR (2008). Excessive adipose tissue infiltration in skeletal muscle in individuals with obesity, diabetes mellitus, and peripheral neuropathy: association with performance and function.. Phys Ther.

[50] Gauthier MS, Miyoshi H, Souza SC, Cacicedo JM, Saha AK (2008). AMP-activated protein kinase is activated as a consequence of lipolysis in the adipocyte: potential mechanism and physiological relevance.. J Biol Chem.

[51] Diamond JM (1982). Transcellular cross-talk between epithelial cell membranes.. Nature.

[52] McKenzie JL, Gan OI, Doedens M, Wang JC, Dick JE (2006). Individual stem cells with highly variable proliferation and self-renewal properties comprise the human hematopoietic stem cell compartment.. Nat Immunol.

[53] Hammond CL, Simbi BH, Stickland NC (2007). In ovo temperature manipulation influences embryonic motility and growth of limb tissues in the chick (Gallus gallus).. J Exp Biol.

[54] Maltby V, Somaiya A, French NA, Stickland NC (2004). In ovo temperature manipulation influences post-hatch muscle growth in the turkey.. Br Poult Sci.

[55] Nesti C, Pasquali L, Vaglini F, Siciliano G, Murri L (2007). The role of mitochondria in stem cell biology.. Biosci Rep.

[56] Jahagirdar BN, Verfaillie CM (2005). Multipotent adult progenitor cell and stem cell plasticity.. Stem Cell Rev.

[57] Mothe AJ, Kulbatski I, van Bendegem RL, Lee L, Kobayashi E (2005). Analysis of green fluorescent protein expression in transgenic rats for tracking transplanted neural stem/progenitor cells.. J Histochem Cytochem.

[58] Milan G, Dalla Nora E, Pilon C, Pagano C, Granzotto M (2004). Changes in muscle myostatin expression in obese subjects after weight loss.. J Clin Endocrin Metab.

[59] Ramirez-Zacarias JL, Castros Munozledo F, Kuri-Harcuch W (1992). Quantitation of adipose conversion and triglycerides by staining intracytoplasmic lipids.. Hystochemistry.

[60] Hall-Craggs EC (1974). Rapid degeneration and regeneration of a whole skeletal muscle following treatment with bupivacaine (Marcain).. Exp Neurol.

