# Supplementary material for: Clonal Characterization of Rat Muscle Satellite Cells: Proliferation, Metabolism and Differentiation Define an Intrinsic Heterogeneity
Source: PLoS One. 2010 Jan 1;5(1):e8523. doi: 10.1371/journal.pone.0008523 (PMC2796166; doi:10.1371/journal.pone.0008523)
Supplement: Table S1 — The primers were built from information taken from: http://frodo.wi.mit.edu/cgibin/primers3/primer3_wwwcgi web site. (0.04 MB DOC) [file pone.0008523.s001.doc]

**Table 1. Primers used for standard and real-time PCR**

The primers were built from information taken from:

<http://frodo.wi.mit.edu/cgibin/>primers3/primer3_wwwcgi web site.

| **Gene** | **Forward** | **Reverse** |
| --- | --- | --- |
| beta-2-microglobulin  (NM 012512) | GCTGTGCTCGCGCTACTCT | CAACTTCAATGTCGGATGG |
| Pax7  (XM 575941) | CACGGTGCCCTCAGTGAGTT | TCTCGCCATCTTCTTCCTTTTT |
| Myf5  (NM  001106783) | GTGTGGAAGACCCCTGGTAA | GCTTTGGCTCATTTTCTGCT |
| MyoD  (NM 176079) | GCTCTGATGGCATGATGGAT | GTGGAGATGCGCTCCACTAT |
| Myogenin  (NM 017115) | ACTACCCACCGTCCATTCAC | TCGGGGCACTCACTGTCTCT |
| UCP-1  (NM 012682) | CACCACACTCCTGGCCTCTC | AAAGAAGGCGCAAACCCTTT |
