# Supplementary material for: Clonal Characterization of Rat Muscle Satellite Cells: Proliferation, Metabolism and Differentiation Define an Intrinsic Heterogeneity
Source: PLoS One. 2010 Jan 1;5(1):e8523. doi: 10.1371/journal.pone.0008523 (PMC2796166; doi:10.1371/journal.pone.0008523)

**Figure S1. Culture of single fibers in suspension in hanging drops**

20 drops with a single myofiber were posed on the top of a petri dish. It was then turned, in order to perform a suspension culture in hanging drops. After 5 days SCs emanate from the fiber and clones were easily distinguishable between LPC and HPC.


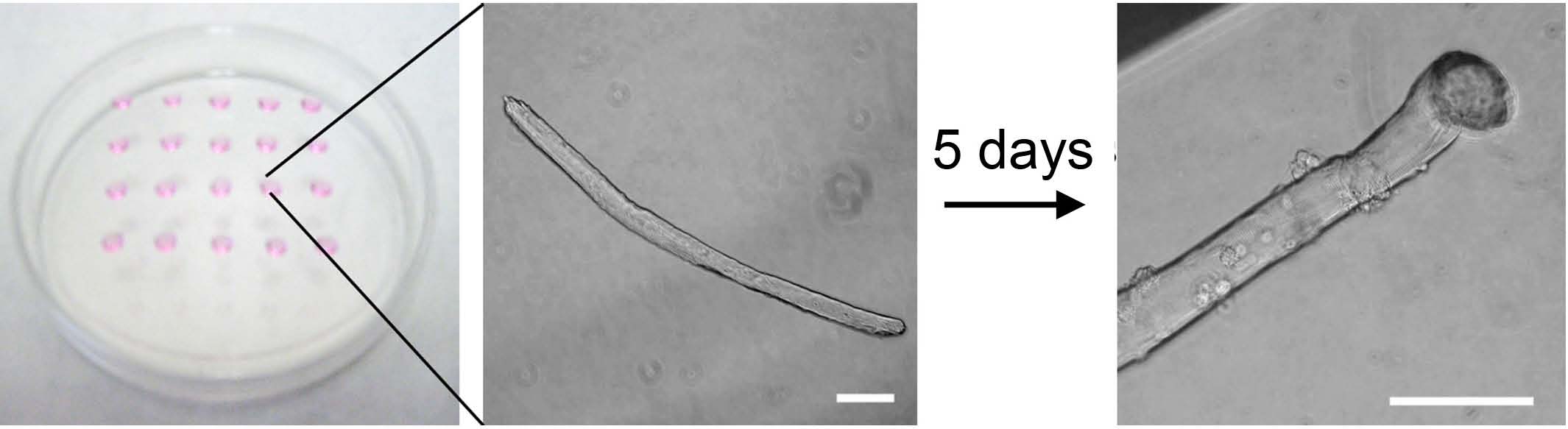

Supplement: Figure S1 — 20 drops with a single myofiber were posed on the top of a petri dish. It was then turned, in order to perform a suspension culture in hanging drops. After 5 days SCs emanate from the fiber and clones were easily distinguishable between LPC and HPC. (0.08 MB DOC) [file pone.0008523.s002.doc]
