# Supplementary material for: Clonal Characterization of Rat Muscle Satellite Cells: Proliferation, Metabolism and Differentiation Define an Intrinsic Heterogeneity
Source: PLoS One. 2010 Jan 1;5(1):e8523. doi: 10.1371/journal.pone.0008523 (PMC2796166; doi:10.1371/journal.pone.0008523)

**Figure S2. Evaluation of SCs number per isolated single fiber before and after fiber disaggregation**

Isolated FDB fibers were immunostained for Pax7 and DAPI. SCs were counted in order to evaluate the average amount present per single, isolated fiber (as in Fig. 1). Diagram shows the average amount of SCs on fiber (F column) and after fiber disaggregation and isolation (AI column, mean ± s.d.).


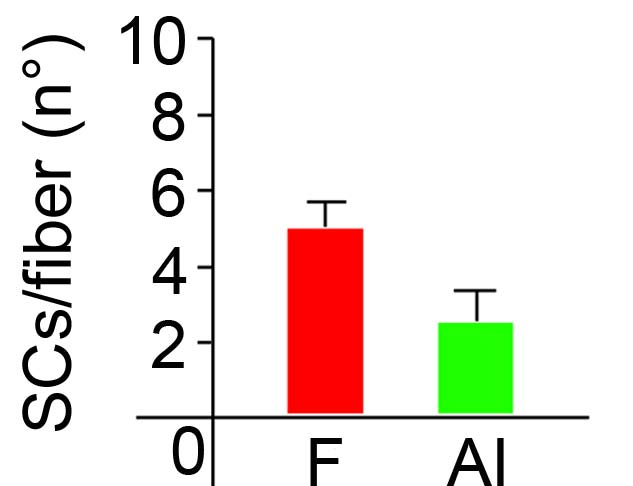

Supplement: Figure S2 — Isolated FDB fibers were immunostained for Pax7 and DAPI. SCs were counted in order to evaluate the average amount present per single, isolated fiber (as in Fig. 1). Diagram shows the average amount of SCs on fiber (F column) and after fiber disaggregation and isolation (AI column, mean±s.d.). (0.04 MB DOC) [file pone.0008523.s003.doc]
