# Supplementary material for: Clonal Characterization of Rat Muscle Satellite Cells: Proliferation, Metabolism and Differentiation Define an Intrinsic Heterogeneity
Source: PLoS One. 2010 Jan 1;5(1):e8523. doi: 10.1371/journal.pone.0008523 (PMC2796166; doi:10.1371/journal.pone.0008523)
Supplement: Figure S3 — SCs released from isolated myofibers were seeded on gelatin-coated slides, and then immunofluorescence for the canonical marker Pax7 and the myogenic markers Myf5 and MyoD were performed. Diagram indicates percentage of positive cells (mean Â±s.d.). (1.03 MB DOC) [file pone.0008523.s004.doc]

**Figure S3. Characterization of freshly isolated SCs**

SCs released from isolated myofibers were seeded on gelatin-coated slides, and then immunofluorescence for the canonical marker Pax7 and the myogenic markers Myf5 and MyoD were performed. Diagram indicates percentage of positive cells (mean ± s.d.).
