# Supplementary material for: Clonal Characterization of Rat Muscle Satellite Cells: Proliferation, Metabolism and Differentiation Define an Intrinsic Heterogeneity
Source: PLoS One. 2010 Jan 1;5(1):e8523. doi: 10.1371/journal.pone.0008523 (PMC2796166; doi:10.1371/journal.pone.0008523)
Supplement: Figure S4 — Freshly isolated SCs were immunostained for haematopoietic cell marker CD45, macrophage marker CD163 and endothelial marker CD31, at each of the 3 passages of dilution (see Materials and Methods). They resulted negative for all contaminant cell markers immediately after the second passage. (15.29 MB DOC) [file pone.0008523.s005.doc]

**Figure S4. Immunostaining for CD45, CD163 and CD31 on preparations of SCs**

Freshly isolated SCs were immunostained for haematopoietic cell marker CD45, macrophage marker CD163 and endothelial marker CD31, at each of the 3 passages of dilution (see Materials and Methods). They resulted negative for all contaminant cell markers immediately after the second passage.
