# Supplementary material for: Clonal Characterization of Rat Muscle Satellite Cells: Proliferation, Metabolism and Differentiation Define an Intrinsic Heterogeneity
Source: PLoS One. 2010 Jan 1;5(1):e8523. doi: 10.1371/journal.pone.0008523 (PMC2796166; doi:10.1371/journal.pone.0008523)
Supplement: Figure S5 — HPC clones (n = 10) were sub-cloned in 96-well dishes with limiting dilutions. After 10 days of culture it was possible to distinguish LPC (left) and HPC (center), the latter with adipogenic potential. Their relative proportion was evaluated and reported in diagram (right, mean Â±s.d., ***p<0.001). In the table below the developmental potential of each single sub-cloning is reported. (0.88 MB DOC) [file pone.0008523.s006.doc]

**Figure S5. Sub-cloning of HPC**

HPC clones (n=10) were sub-cloned in 96-well dishes with limiting dilutions. After 10 days of culture it was possible to distinguish LPC (left) and HPC (center), the latter with adipogenic potential. Their relative proportion was evaluated and reported in diagram (right, mean ± s.d., ***p<0.001). In the table below the developmental potential of each single sub-cloning is reported.

| **HPC clone (n°)** | **LPC clones** | **HPC clones** |
| --- | --- | --- |
| 1 | 115 | 0 |
| 2 | 107 | 0 |
| 3 | 118 | 7 |
| 4 | 109 | 11 |
| 5 | 105 | 13 |
| 6 | 92 | 23 |
| 7 | 91 | 25 |
| 8 | 97 | 28 |
| 9 | 84 | 36 |
| 10 | 85 | 39 |
