# Supplementary material for: Clonal Characterization of Rat Muscle Satellite Cells: Proliferation, Metabolism and Differentiation Define an Intrinsic Heterogeneity
Source: PLoS One. 2010 Jan 1;5(1):e8523. doi: 10.1371/journal.pone.0008523 (PMC2796166; doi:10.1371/journal.pone.0008523)
Supplement: Figure S6 — When the number of Pax7+/GFP+ cells was evaluated after LPC and HPC injection we could not observe any significant difference as reported in the diagram (mean Â±s.d.). (0.06 MB DOC) [file pone.0008523.s007.doc]

**Figure S6. Pax7+/GFP+ cells**

When the number of Pax7+/GFP+ cells was evaluated after LPC and HPC injection we could not observe any significant difference as reported in the diagram (mean ± s.d.).
